# Biomarker patterns reflecting habitual food intake: the BARITONE sample and randomized controlled crossover trial protocol

**DOI:** 10.3389/fnut.2026.1977025

**Published:** 2026-10-29

**Authors:** Lisa-Maria Oberrauter, Helen M. Lindqvist, Anna Winkvist

**Affiliations:** Department of Internal Medicine and Clinical Nutrition, Institute of Medicine, Sahlgrenska Academy, University of Gothenburg, Gothenburg, Sweden

**Keywords:** dietary assessment, dietary biomarkers, fruits and vegetables, metabolomics, nutrition epidemiology, protein intake

## Abstract

**Background:**

Accurate assessment of dietary intake remains a major challenge in nutrition research and limits the ability to study diet–disease relationships. Most epidemiological studies rely on self-reported dietary data that are subject to measurement errors. Metabolomics has emerged as a promising approach for identifying objective biomarkers of dietary intake. However, validated biomarkers for current intake are still lacking for several key food groups, including different protein sources and vegetables, fruits, and berries.

**Objective:**

The objective of this study is to identify and validate metabolomic biomarker patterns reflecting intake of these food groups as well as to identify groups of individuals with different responses to the trial foods.

**Methods:**

The BARITONE project consists of two parts. First, individuals were recruited to a screening session. Second, from this larger sample, a subset of participants with high and low, respectively, intake of vegetables, fruit and berries were selected for participation in a randomized controlled three-period crossover dietary intervention trial. In the trial, participants consumed three dietary patterns differing in primary protein source (meat-based, fish-based, and plant-based) during three 1-week intervention periods separated by 1-week washout periods. Fasting blood and urine samples were collected at screening and pre- and post- each intervention week. Habitual dietary intake was assessed using 7-day weighed food diaries collected at screening and during both washout periods. Metabolomic profiling will be applied to identify biomarker patterns associated with the source of protein as well as changes in intake of vegetables, fruit and berries. These patterns of metabolites will be confirmed in the data on habitual dietary intake from the screening sample.

**Discussion:**

This study provides a framework for identifying and validating metabolomic biomarker patterns of present dietary intake by combining controlled and observational data. The findings may contribute to improved dietary assessment and support future research on diet–health relationships.

**Clinical trial registration:**

NCT07128940.

## Introduction

Diet is a major modifiable determinant of chronic disease risk and is among the leading contributors to premature mortality worldwide (1–3). Suboptimal dietary patterns, characterized by high intake of red and processed meat, sodium, and trans fatty acids, and low intake of fruits, vegetables, and plant-based foods, are associated with increased risk of cardiovascular disease, cancer, and other non-communicable diseases (1, 2). In addition to health impacts, current dietary patterns also have significant environmental consequences, as food production contributes substantially to global greenhouse gas emissions, with animal-based foods—particularly red meat—representing a major source (2). As a result, dietary shifts toward more plant-based and diverse dietary patterns are increasingly emphasized for both human health and environmental sustainability.

Despite the central role of diet in health and sustainability, accurately measuring dietary intake remains a major challenge in nutrition research. Most epidemiological studies rely on self-reported dietary assessment methods, such as food frequency questionnaires and dietary records, which are subject to both random and systematic measurement errors (4). At the individual level, these include intake-related as well as person-specific bias. Examples of intake-related bias are underreporting, recall bias, and difficulties in estimating portion sizes and total intake. Person-specific bias includes day-to-day variation in food intake, variability in food composition and processing variations of food items (4). On a structural level errors include lack of suitable food items and low precision in food composition tables. Such limitations can lead to misclassification of dietary exposure and attenuation of associations between diet and health outcomes, thereby reducing the reliability of findings and limiting the ability to draw robust conclusions about diet–disease relationships (5, 6). Improving the accuracy of dietary assessment is therefore a key priority for advancing nutrition science and informing effective public health strategies.

In recent years, metabolomics has emerged as a promising approach for improving dietary assessment through the identification of objective biomarkers of food intake (6–8). Metabolomics involves the comprehensive analysis of low-molecular-weight metabolites in biological samples such as blood and urine. These metabolites reflect both exogenous compounds derived from food intake and endogenous metabolic processes influenced by host and microbiome interactions. As such, the metabolome provides a sensitive and integrative measure of an individual’s metabolic phenotype at a given time point. Compared with traditional dietary assessment methods, metabolomics-based approaches have the potential to reduce measurement error and improve the precision of dietary intake estimates. In addition, the use of metabolomic patterns rather than single biomarkers may better capture the complexity of dietary exposures and improve the ability to distinguish between different dietary patterns (7, 9). However, translation of metabolomic findings into validated dietary biomarkers remains limited.

Validated biomarkers are still lacking for several key food groups that are central to dietary recommendations and health outcomes, including different sources of dietary protein (e.g., meat, fish, and plant-based foods) as well as vegetables, fruits, and berries (6, 9, 10). Identifying biomarkers for these food groups presents distinct methodological challenges. For protein sources, well-controlled feeding studies are required to isolate metabolic responses to specific dietary exposures and to distinguish between different protein types under standardized conditions. In contrast, for vegetables, fruits, and berries, biomarker identification is more complex, as low intake does not correspond to a specific alternative exposure but rather reflects a wide range of dietary patterns in free-living populations.

To address these challenges, recent work has emphasized the importance of combining controlled feeding studies with observational data. Controlled dietary interventions are essential for identifying metabolomic signatures of recent intake under standardized conditions, while observational studies are required to evaluate the robustness, reliability, and generalizability of these biomarkers in habitual dietary contexts (10, 11). Criteria for biomarker validation have been proposed, including evaluation of plausibility, dose–response relationships, time-response, robustness, and reliability across study settings (10).

The BARITONE project was developed to address these methodological challenges by integrating an observational study with a controlled dietary crossover trial. This combined design enables identification of metabolomic biomarker patterns under controlled conditions and their validation in relation to habitual dietary intake and inter-individual variability in real-world settings.

### Study aim

The objectives of the BARITONE project are to (1) identify and validate metabolomic biomarker patterns reflecting intake of different protein sources, (2) identify and validate metabolomic patterns associated with high and low intake of vegetables, fruits, and berries, and (3) identify metabotypes based on responses to the dietary exposures.

## Materials and methods

### Trial design

The BARITONE project consists of two components. First, the BARITONE sample constitutes a cross-sectional study in which participants undergo comprehensive baseline screening with extensive phenotyping, including a 7-day weighed food diary. Second, the BARITONE trial includes a selected subgroup from the BARITONE sample who participate in a randomized controlled three-period crossover dietary intervention study. The BARITONE project was registered at ClinicalTrials.gov on 2025-06-26 (NCT07128940). The target sample size is 70 participants, with 60 expected to complete all three periods. Participants consume three dietary patterns (meat-based, fish-based, and vegetarian) in randomized order. Each intervention period lasts 1 week and is separated by a 1-week washout period to minimize potential carryover effects. The washout periods are further used to identify putative biomarker patterns associated with changes in intake levels of vegetables, fruits, and berries. Intake of vegetables, fruits, and berries is standardized across all three intervention diets. Participants habitually consume varying amounts of vegetables, fruits, and berries, and the majority will either increase or decrease their intake during the intervention periods, relative to the screening and washout periods that mirror habitual intake.

The schedule of enrolment, interventions, and assessments is presented according to Standard Protocol Items: Recommendations for Intervention Trials (SPIRIT) in Figure 1. The SPIRIT Checklist is provided in Supplementary Table S1.

**Figure 1:**
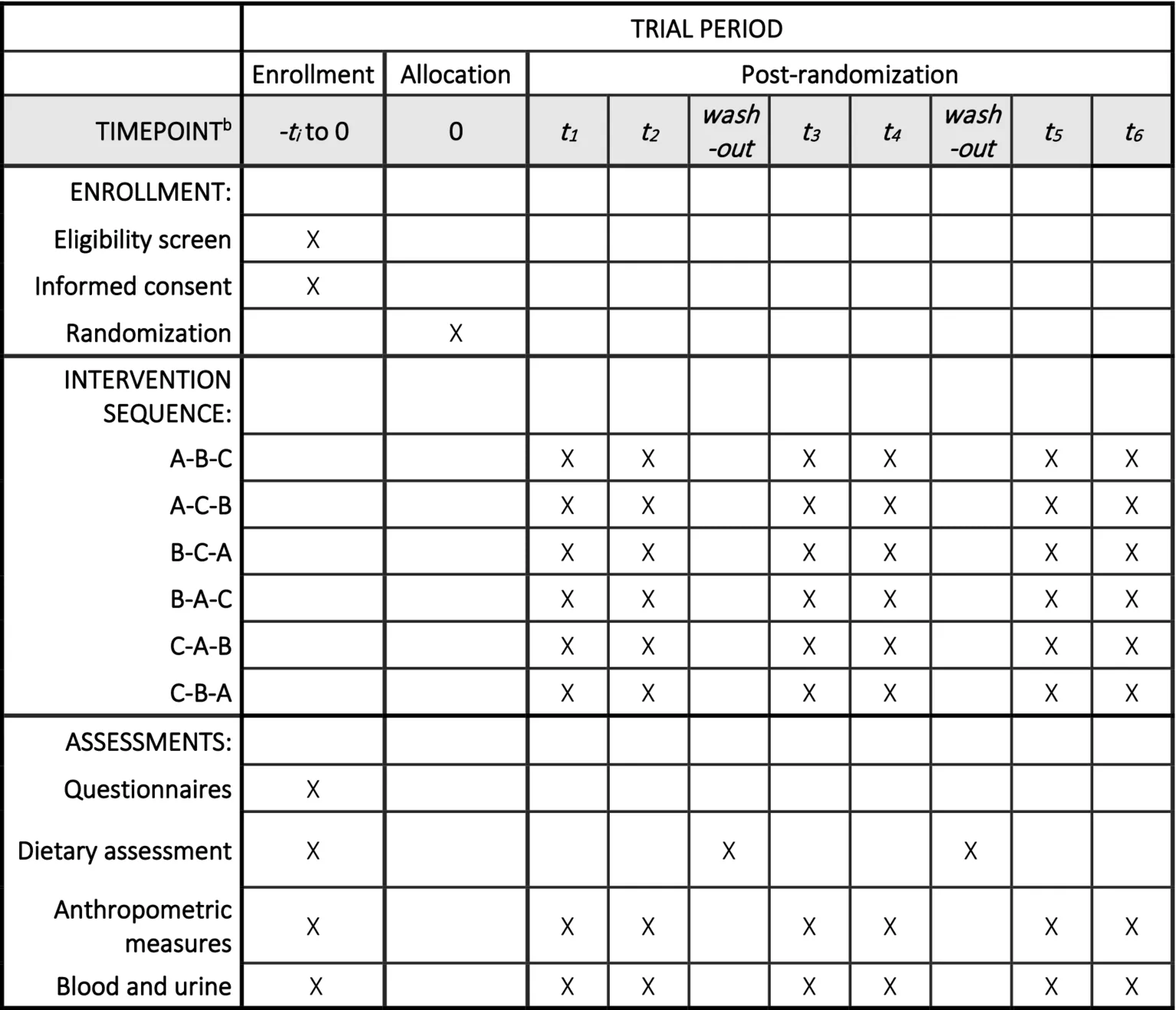
Participant timeline: Schedule of enrolment, interventions, and assessments for the BARITONE project.

### Trial setting

The baseline screening and the intervention trial are conducted at the Metabolic Kitchen and Laboratory, the Department of Internal Medicine and Clinical Nutrition, Sahlgrenska Academy, University of Gothenburg, Gothenburg, Sweden. Participants attend study visits at the laboratory facilities, while the dietary interventions are carried out under free-living conditions.

### Recruitment

Participants are being recruited since fall 2025 through advertisements on recruitment platforms, social media, and university campuses in Gothenburg, Sweden. Recruitment materials provide information about the study aims, eligibility criteria, and contact details for the research team. Pre-screening is conducted using an online questionnaire to identify potentially eligible individuals. Recruitment to the BARITONE trial continued until the predefined target sample size (*n* = 70) was reached.

### Participant selection and eligibility criteria

The pre-screening online questionnaire includes socio-demographic and lifestyle questions covering chronic illnesses, pharmacological treatment, and physical activity, as well as a short dietary intake assessment using a food frequency questionnaire (FFQ) targeting vegetables, fruits, and berry intake. Eligible candidates (*n* = 287) are invited to attend the baseline screening at the research facilities and are instructed to complete a 7-day weighed food diary during the week prior to their appointment.

At the baseline screening, study details are presented orally and in writing and written informed consent is obtained by study personnel. Participants are asked to complete an extended FFQ and lifestyle questionnaire. Measurements of body weight and height are performed, as well as body composition assessed by bioelectrical impedance analysis (BIA). Fasting venous blood samples are taken by a registered nurse and fasting spot urine samples are collected. Further, participants provide a frozen stool sample collected prior to the visit.

Adults who consider themselves healthy, aged 18–65 years with a body mass index (BMI) of 18.5–30 kg/m^2^ are eligible for participation. Participants are required to be willing to consume all three dietary patterns (meat-based, fish-based, and vegetarian diets) and to understand both written and spoken Swedish. Exclusion criteria include pregnancy or lactation; cancer or other life-threatening diseases; and metabolic or diet-related disorders such as diabetes, inflammatory bowel disease, hypothyroidism, or celiac disease. Individuals with allergies or intolerances that prevented consumption of foods included in the intervention diets are also excluded. Furthermore, the use of medications known to affect metabolism (e.g., lipid-lowering agents or insulin), as well as the use of illicit drugs, alcohol, or nicotine products (including smoking, snus, or vaping), constitute exclusion criteria.

Participants meeting the eligibility criteria are subsequently considered for inclusion in the BARITONE trial (*n* = 70). An equal distribution by sex and varying intake levels of vegetables, fruits, and berries has been prioritized. The latter was done to address the aim of identifying biomarker patterns related to changes in intake of vegetables, fruits, and berries. High intake (highest tertile, estimated >500 g/day) or low intake (lowest tertile, estimated <200 g/day) is based on intake data from the Swedish national dietary intake survey Riksmaten 2010–2011 (12) and assessed using the pre-screening questionnaire (FFQ). The diet intervention will be delivered by a registered dietician.

### Randomization

After inclusion, participants are randomized to the order of the trial diets using a computer-generated allocation sequence. The randomization scheme was determined by an external personnel by Excel randomization function using a computer-generated allocation sequence. Participants are assigned to diet sequences in a 1:1:1 ratio to ensure balanced distribution of intervention orders. Blinding of participants or staff is not possible in this diet intervention.

### Diet intervention

In the BARITONE trial, participants follow three 1-week dietary intervention periods, each dietary week separated by a 1-week long washout period (Figures 1, 2). The intervention weeks consist of predefined daily meal plans including breakfast, lunch, dinner, and snacks, with the primary difference between them being the protein source used in lunch and dinner meals (meat, fish, or plant-based alternatives). The overall meal structure and nutritional composition are kept comparable across all conditions. During the washout weeks, participants are instructed to resume their habitual diet and to record their intake using detailed 7-day weighed food diaries.

**Figure 2:**
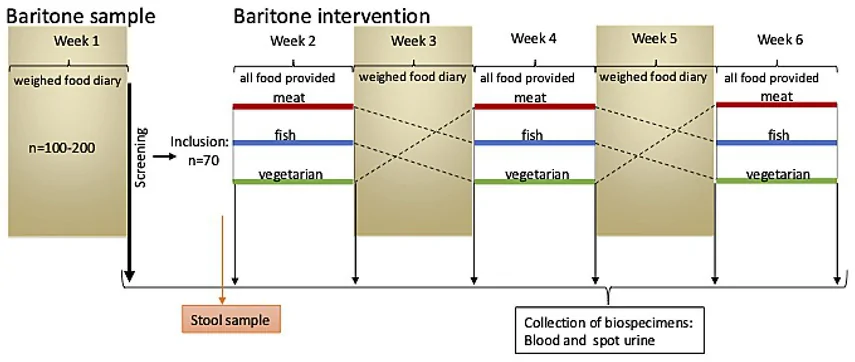
Flow chart of BARITONE sample and dietary crossover intervention.

### Diet composition and nutritional targets

The meal plans were designed to be comparable across intervention conditions with respect to total energy intake and macronutrient distribution (Table 1). Two predefined energy levels are used based on the Nordic Nutrition Recommendations [NNR 2023 (13)]: energy level 1 [9.0 MJ/day; 2,151 kcal/day, ±5% (2,043–2,259 kcal/day)] and energy level 2 [11.3 MJ/day; 2,701 kcal/day, ±5% (2,566–2,836 kcal/day)], corresponding to the recommended energy intake for women and men aged 25–50 years with moderate physical activity (PAL 1.6), respectively. The macronutrient composition follows the recommended ranges according to NNR 2023, corresponding to 45–60 energy percent (E%) from carbohydrates, 10–20 E% from protein, and 25–40 E% from fat.

**Table 1:** Energy and macronutrient composition of diets in the BARITONE crossover trial^a.^

Macronutrient	Meat diet		Fish diet		Vegetarian diet	
	Energy level 1	Energy level 2	Energy level 1	Energy level 2	Energy level 1	Energy level 2
Energy (kJ)	8,779	11,151	8,770	11,079	8,824	11,208
Energy (kcal)	2096	2,663	2,100	2,652	2,117	2,681
Carbohydrate						
g	240	297	232	289	246	305
E %	48	47	47	46	50	48
Protein						
g	94	125	94	124	88	116
E %	18	19	18	19	17	18
Fat						
g	78	101	81	103	78	100
E %	33	34	35	35	33	34
Vegetables/fruit/berries (g/day)	364	364	361	361	364	364

^a^Values represent planned daily intake based on weekly averages. Macronutrient distribution is expressed as grams (g) and percentage of total energy intake (E%).

The total intake and types of vegetables, fruits, and berries was predefined and kept consistent across intervention conditions at approximately 350 g/day, expressed as a weekly average, corresponding to the median intake reported among Swedish adults in Riksmaten 2010–2011 (12).

Allocation to energy level is based on individual estimated energy requirements derived from Seca-calculation, i.e., based on sex, age, BMI and self-reported physical activity level (PAL). PAL is estimated using the model of Johansson and Westerterp (14) from two questions on physical activity at work and leisure time included in the pre-screening questionnaire.

### Meal structure and menu design

Menus were developed in advance by a registered dietitian (LMO) and followed a fixed 7-day structure for each intervention period. Each daily menu consists of three main meals (breakfast, lunch, and dinner) and two snacks. Meals are standardized across conditions, with the same recipes used whenever possible. The protein source at lunch and dinner differs between conditions (meat, fish, or plant-based alternatives), whereas breakfast and snacks are identical across all conditions. Apart from these substitutions, meal components, side dishes, and overall menu structure are kept consistent. Minor adaptations to recipes are made for the fish-intervention week to ensure palatability and participant acceptance; however, overall energy content, macronutrient distribution, and meal composition are kept comparable. Recipes were designed to be simple to prepare, culturally accepted, and based on commonly consumed foods to support participant adherence to the intervention. Table 2 shows an example of a standardized daily menu across intervention weeks differing only in protein source.

**Table 2:** Example of standardized daily menu across intervention weeks differing only in protein source.

Meal	Meat	Fish	Vegetarian
Breakfast	Porridge apple sauce dried cranberries almonds	Porridge apple sauce dried cranberries almonds	Porridge apple sauce dried cranberries almonds
Snack 1	Sandwich with cheese and cucumber	Sandwich with cheese and cucumber	Sandwich with cheese and cucumber
Lunch	Beef Stroganoff rice	Salmon Stroganoff rice	Soy Stroganoff rice
Snack 2	Pear muesli bar	Pear muesli bar	Pear muesli bar
Dinner	Wok with rice noodles, beef and vegetables	Wok with rice noodles, cod and vegetables	Wok with rice noodles, tempeh and vegetables

### Intervention delivery

The dietary intervention is delivered through provision of food, recipes, and detailed instructions. General instruction, ingredient list, recipes and the weekly menu are provided in a bag together with breakfast, lunch, and snacks for the first day of each intervention week at the study visit. All remaining ingredients required for the intervention period are delivered to participants’ homes via an online grocery delivery service on the day prior to the start of each intervention period. Prespecified food products are used throughout the intervention whenever available, and any necessary substitutions are documented. Meals are prepared by participants using standardized recipes, specified quantities and detailed preparation instructions to minimize variation in food composition and preparation. Because recruitment and data collection extend over time, foods are not necessarily obtained from the same production batch. A summary of the provided foods, recipes, portion sizes and preparation instructions and nutrient composition will be published together with the intervention results.

### Participant instructions and adherence

Participants receive written instructions to support adherence to the dietary intervention. They are asked to follow the prescribed daily menus and to prepare meals according to the provided recipes and portion sizes corresponding to their assigned energy level. Participants are instructed to consume all components of the meals, including prescribed amounts of vegetables, fruits, and berries. If meals cannot be completed in full, they are advised to prioritize consumption of the main protein component and vegetables, fruits and berries, and to adjust carbohydrate intake as needed to achieve satiety while maintaining weight stability. Some flexibility is allowed, including variation in the timing and order of meals. Participants are advised to avoid consumption of non-study foods except for water, coffee, and tea without added sugar, and a very limited intake of sweets and snacks according to predefined guidelines. Adherence to the intervention is assessed using a deviation report completed by participants throughout each intervention period to record any departures from the prescribed diet. The deviation report includes the amounts of provided foods not consumed and any non-study foods consumed, where applicable.

### Discontinuation and modification of the intervention

Participants can discontinue the intervention at any time upon request. Discontinuation may also occur if participants are unable to adhere to the study protocol or if any adverse events or intolerances related to the intervention food are reported.

### Concomitant care

Participants are instructed to maintain their usual lifestyle habits, including physical activity and sleep patterns, throughout the study period. As the study population consists of adults, regarding themselves as healthy, regular use of medications is not expected, and participants are asked to inform the study team if any new medications are initiated during the study. Habitual dietary supplements are allowed during the study; however, the use of creatine monohydrate is not permitted, as this may influence metabolomic profiles related to amino acid and protein metabolism.

### Harms

Given the short duration of the dietary interventions and the use of commonly consumed foods, the risk of adverse events is considered low. Potential harms may include mild transient gastrointestinal discomfort due to dietary changes, sudden allergic reactions or food intolerances, and minor discomfort associated with blood sampling (e.g., bruising or dizziness). Adverse events are assessed non-systematically through participant self-report during study visits and through communication with the study team throughout the intervention period i.e., per e-mail or phone. Any reported adverse events are documented and evaluated by the investigators.

### Data collection and outcome assessment

Data are collected at screening visit and throughout each intervention period according to a standardized schedule. Biological samples and anthropometric measurements are obtained at the beginning and end of each intervention period.

#### Biological sample collection and processing

Fasting blood samples are collected at screening and before and after each intervention period (up to seven time points for participants completing all intervention periods). Blood lipids, hemoglobin (Hb), glycated hemoglobin (HbA1c), thyroid hormones, and high-sensitivity C-reactive protein (hsCRP) are analyzed in fresh samples at the laboratory of Clinical Chemistry, Sahlgrenska University Hospital, Gothenburg, Sweden, following standard procedures.

Venous blood is drawn into EDTA, lithium heparin, and serum tubes and processed according to standardized procedures, including centrifugation and aliquoting. Samples are initially stored at −20 °C and subsequently transferred to −80 °C as soon as possible (within 24 h), where they are stored until analysis.

Fasting urine samples are collected and kept refrigerated for a maximum of 2 h before being centrifuged and aliquoted. Samples are then initially stored at −20 °C and subsequently transferred to −80 °C within 24 h, where they are stored until analysis.

#### Dietary assessment

Dietary intake during washout periods is assessed using 7-day weighed food diaries. Participants receive written instructions on how to record all foods and beverages consumed. The instructions encourage the use of a kitchen scale to improve accuracy, but also include pictures for estimating portion sizes. Completed food diaries are reviewed for completeness, and participants are contacted if clarification is required.

Habitual intake of vegetables, fruits and berries (g/day) will be calculated from screening food diaries for all participants and categorized into high intake (>500 g/day), medium intake (200–500 g/day) or low intake (<200 g/day) groups. In addition, habitual intake of meat, fish, and vegetarian protein sources will be calculated for all food diaries.

#### Anthropometric measurements

At screening, height (using a stadiometer), waist- and hip-circumference are measured. A bioelectrical impedance analysis device (Seca mBCA 515/514, Seca analytics 115, Seca, Germany) is used to measure weight and body composition. Weight is measured using the same device at all study visits.

#### Data quality and standardization

All measurements are conducted by trained research staff following standardized protocols. Data collection procedures are consistent across all intervention periods. Participant-reported data and study records are checked for completeness and plausibility during each visit, and discrepancies are resolved where possible.

#### Metabolomics and lipidomics

Plasma metabolomic and lipidomic profiling will be performed at the Swedish Metabolomics Center, Umeå, Sweden. Metabolites will be extracted using methanol–water and analyzed by gas chromatography–time-of-flight mass spectrometry (GC–TOF–MS) and ultra-high-performance liquid chromatography–quadrupole time-of-flight mass spectrometry (UHPLC–QTOF–MS). Lipids will be extracted using a modified Folch protocol and analyzed by UHPLC–QTOF–MS in positive ion mode. Samples will be analyzed in randomized run order, with internal standards, extraction blanks and pooled quality control samples included to monitor analytical quality. Metabolites and lipids will be annotated using reference libraries. The LC–MS platform generates some 1,600 untargeted and 300 targeted metabolites and the GC–MS platform about 80 tentatively annotated metabolites. Both unsupervised and supervised analyses will be performed.

To ensure analytical quality, samples will be analyzed in randomized run order. Stable-isotope-labeled internal standards, extraction blanks and pooled quality-control (QC) samples injected at regular intervals will be used to monitor sample preparation and analytical performance. QC dilution series will additionally be used to assess signal linearity where applicable. Accurate mass measurements will be supported by continuous internal mass calibration, and analytical features will be filtered based on blank levels and QC performance. Full platform-specific procedures will be reported with the intervention results.

#### Microbiota

16S ribosomal gene sequencing of stool samples will be carried out at Umeå University, Umeå, Sweden, with Nanopore Sequencing, developed by Oxford Nanopore Technologies (ONT). The method sequences the entire 16S gene, yielding high precision taxonomy. The method is further described in Esberg et al. (15).

### Analysis of outcome

Primary outcome of the trial is differences in patterns of metabolites in plasma at the end of the three intervention weeks. Metabolite pattern changes across the three intervention weeks will also be compared between participants with habitual high and low intake of vegetables, fruits and berries. Secondary outcomes include changes at the end of the three intervention weeks in patterns of urine metabolites, and lipids (lipidomics) in plasma. We expect patterns in plasma and urine to be complementary, rather than interchangeable. Metabolite patterns in plasma are chosen as primary outcome because of challenges with wide ranges of urine concentrations and uncertainty about the optimal timing of sampling spot urine for detecting various food metabolites. Also, in large cohort studies serum samples are more commonly collected than are urine samples. Hence, results on the former as potential objective biomarkers of intake may be more useful for the research community in nutritional epidemiology (see Supplementary Table S2 for details about outcome measurements).

Physical activity level, body composition, weight, BMI, triacylglycerides, total cholesterol, LDL, HDL, Apolipoprotein A1 and B and microbiota from stool samples, will be analyzed at baseline for descriptive purposes. Hb, HbA1c, thyroid hormones, and hsCRP were evaluated at inclusion to ensure a healthy study sample.

### Data analysis and biostatistics

Unsupervised Principal Component Analysis (PCA) and supervised approaches based on Orthogonal Partial Least Squares (OPLS) will be used to characterize plasma and urine metabolomic patterns and lipidomic patterns associated with dietary intake. The primary comparison will concern plasma metabolomic patterns at the end of the three diet intervention periods. Orthogonal partial least squares hierarchical discriminant analysis (OPLS-HDA) will be used for the overall comparison among the three diets intervention periods. End-of-period profiles, rather than within-period changes, will be used for this comparison because the aim is to characterize profiles associated with intake during each intervention period, rather than changes relative to the preceding dietary and metabolic state. OPLS-HDA does not explicitly account for within-participant dependence or adjust for period-specific pre-intervention profiles. Its results will therefore be interpreted as an exploratory overview of profile discrimination, and loadings and variable importance measures will not be interpreted as estimates of individual metabolite intervention effects. The three pairwise comparisons will thereafter be conducted using orthogonal partial least squares effect projections (OPLS-EP) for paired samples, with adjustment for multiple comparisons. Inference regarding intervention effects will rely on the paired analyses and mixed models accounting for participant and period.

Within-period changes in metabolomic patterns will also be compared among participants with different expected changes in their intake of vegetables, fruit, and berries during the intervention. Habitual total intake will be calculated from the 7-day weighed food diary collected at screening and compared with the 350 g/day provided during each intervention week. The percentage difference relative to the provided intake will be calculated as 100 × (350 − habitual intake)/350, with habitual intake expressed in g/day. For OPLS-HDA analyses, participants will be classified as expected to increase intake (≥30%), to decrease intake (≤ − 30%) or to remain relatively stable (> − 30% and <+30%). Because vegetables, fruits and berries represent a heterogeneous food category, exploratory analyses may also examine vegetables, fruits and berries separately. Where the dietary records and intervention menus demonstrate sufficient exposure and variation, biologically or chemically meaningful food subgroups, such as citrus fruits, or individual foods will be explored. These groupings will be defined based on the dietary intake data before examination of the metabolomics results.

Separate linear elastic net models will use within-period metabolomic changes as predictors and the expected change in intake in g/day as the continuous outcome, separately for plasma and urine, following the model-development and internal-validation procedures described below. For these models, the expected change will be calculated for each participant and intervention period as 350 g/day minus the mean daily intake recorded during the 7 days immediately preceding that intervention period. The continuous values will be used without categorization. As a complementary analysis, associations between the continuous expected change in intake (g/day) and within-period changes in individual selected metabolites will be evaluated using mixed models accounting for participant and period, with adjustment for multiple testing.

Potential residual carryover will be explored using OPLS-HDA by comparing metabolomic profiles obtained before the first dietary intervention period with profiles obtained at the end of each of the two subsequent one-week washout periods (i.e., beginning of subsequent dietary intervention period). Post-washout profiles will be grouped according to the preceding dietary intervention. Differences in these metabolic profiles will be interpreted as exploratory evidence.

The primary intervention analyses will follow the randomized diet assignments and use available outcome data, irrespective of adherence. Missing outcomes will not be imputed and only individuals with complete sets for the specific analysis will be included, i.e., for OPLS-HDA all periods and for each OPLS-EP comparison both periods included in that comparison. The number of participants and observations included in each analysis will be reported.

For exploratory PCA and OPLS analyses, variables will be scaled to unit variance. PCA will be used to explore clustering and identify potential outliers. OPLS model robustness will be evaluated using participant-level cross-validation, analysis of variance of cross-validated residuals (CV-ANOVA) and 999 permutation tests. All samples and intervention periods from the same participant will be assigned to the same cross-validation group. Model fit and cross-validated predictive ability will be summarized using R^2^ and Q^2^, respectively. Metabolites contributing most strongly to group discrimination will be ranked using model loadings and variable-importance measures and subsequently evaluated in exploratory analyses of individual metabolites. The Human Metabolome Database (HMDB) will support assessment of the biological and chemical plausibility of candidate biomarkers. Individual metabolites will also be evaluated using mixed models with participant as a random effect and diet, intervention period and randomized diet sequence as fixed effects.

Separate predictive biomarker models will be developed for each dietary protein source using linear elastic net regression, with assigned intake expressed in g/day as the outcome. End-of-period metabolomic profiles from all three dietary intervention periods will be included, with zero intake assigned to periods in which the corresponding protein source is not provided. Models will be developed separately for plasma and urine. Elastic net will select predictors directly from the metabolomic variables, without prior outcome-based selection using OPLS results, correlations, significance tests or univariable discrimination thresholds. Model development and internal validation will be performed using repeated nested participant-level cross-validation. All samples from each participant will be retained within the same fold. Within each outer training set, inner participant-level cross-validation will be used to tune both the elastic net mixing parameter and the regularization strength. All data-dependent preprocessing, including transformation, scaling, imputation of missing metabolite values, if applicable, and feature filtering, will be fitted within the relevant training folds in both the inner and outer loops and applied unchanged to the corresponding held-out data. The complete nested cross-validation procedure will be repeated using different prespecified random fold assignments to assess variability in model performance and metabolite selection.

Internally validated predictive performance will be estimated exclusively from predictions for participants held out in the outer cross-validation loop. Performance will be evaluated using root mean squared error, mean absolute error and the coefficient of determination. Biomarker-selection stability will be assessed by reporting the frequency with which individual metabolites receive non-zero coefficients across resamples. Following internal validation, a final model will be fitted using all eligible intervention participants. Performance estimates obtained from the data used to fit this final model will not be interpreted as validated performance.

Following model development and internal validation using intervention data, the final protein-source models will first be applied without re-estimation to screening metabolomic profiles from the same intervention participants, using their 7-day weighed food diaries as the reference dietary assessment method. This exploratory evaluation will assess performance under habitual dietary conditions but will not constitute independent validation because these participants contributed to model development. The locked protein-source models will subsequently be evaluated in approximately 30 additional eligible BARITONE screening participants who did not enter the intervention and will not contribute to model development or tuning.

Sex, age, body mass index (BMI) and habitual dietary intake will be compared descriptively between the development and validation samples. Given the limited size of the separate BARITONE validation sample, predictive performance estimates from this sample will be interpreted as preliminary and reported with confidence intervals. Further validation in independent cohorts will be required.

Concordant and complementary findings from plasma and urine will be evaluated after separate model development and validation in each matrix (Figure 3). Subsequent external validation of the protein-source models is planned in the VOV (Vegans, Omnivores and Vegetarians) observational study (16, 17), using serum and urine metabolomics data and a 4-day weighed food record as the reference dietary assessment method. The compatibility of analytical measurements and predictors will be assessed before applying the locked models. These evaluations will assess robustness and reliability of the results by comparing controlled dietary exposures to measures of habitual intake.

**Figure 3:**
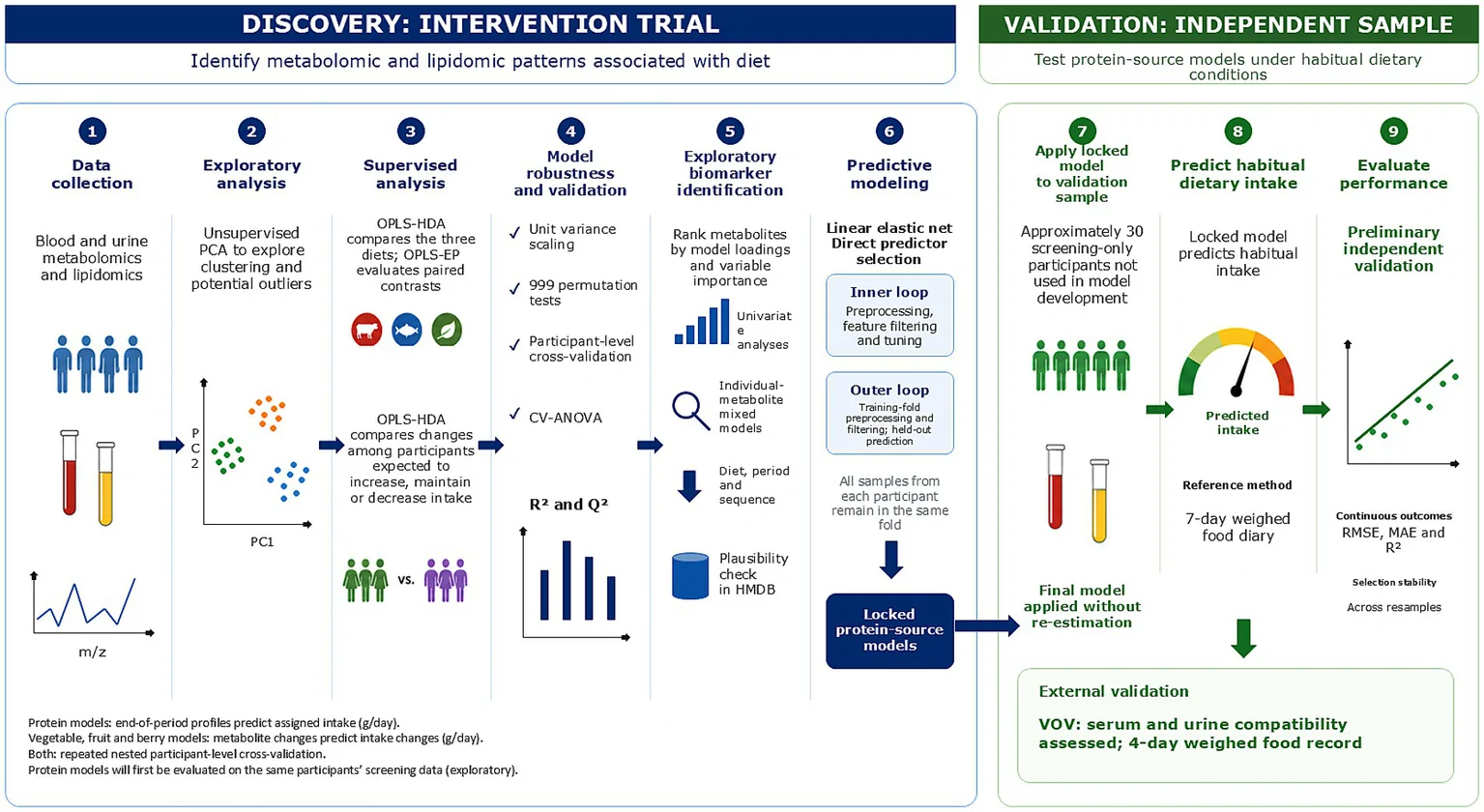
Overview of the planned biomarker discovery, model development and validation in BARITONE. Exploratory analyses and predictive modeling will be conducted separately for plasma and urine. Independent screening validation and external validation will concern the protein-source models. Abbreviations: PCA, principal component analysis; PC1 and PC2, first and second principal components; OPLS-HDA, orthogonal partial least squares hierarchical discriminant analysis; OPLS-EP, orthogonal partial least squares effect projections; CV-ANOVA, analysis of variance of cross-validated residuals; HMDB, Human Metabolome Database; RMSE, root mean squared error; MAE, mean absolute error; *R*^2^, coefficient of determination; Q^2^, cross-validated predictive ability; VOV, Vegans, Omnivores, and Vegetarians; m/z, mass-to-charge ratio; g/day, grams per day.

In sensitivity analyses, per-protocol analyses will be carried out among participants with high adherence. For the protein-source interventions, high adherence for each period is defined as consuming ≥90% of the provided meat, fish or vegetarian protein, consuming no additional protein source of this kind (meat, fish, vegetarian protein sources, egg, protein drinks) during intervention weeks, and remaining weight stable (an absolute weight change of <2% during an intervention week). For vegetables, fruit, and berries, high adherence is defined as consuming at least 90% of the provided amount and no more than 35 g/day of additional vegetables, fruit or berries.

Variation in consumed amounts and documented protocol deviations will be addressed in the per-protocol sensitivity analyses, whereas unmeasured variation in food composition and preparation cannot be incorporated directly into the prediction models.

Exploratory analyses will investigate heterogeneity in metabolic responses to the BARITONE interventions. Within-period changes in metabolite concentrations will be calculated for each participant and dietary intervention. Following unit-variance scaling, PCA will be used for dimensionality reduction, and hierarchical clustering using Ward’s method and Euclidean distance will be applied to the retained component scores to explore potential metabolic response groups (metabotypes). The number of clusters and their stability will be evaluated using cluster-separation measures and participant-level resampling. Associations of the resulting groups with clinical characteristics, gut microbiome profiles and habitual diet will subsequently be explored.

### Sample size and justification

Determining an appropriate sample size for the BARITONE project was challenging due to the exploratory nature of metabolomic biomarker discovery. Previous studies have reported significant associations between metabolite patterns and dietary intake in samples of 98 participants (18). In addition, metabolomic prediction models developed in small samples (*n* = 19) have been successfully validated in independent cohorts of up to 285 participants (19), suggesting that meaningful metabolomic patterns can be identified in relatively small populations. Based on these considerations, a sample size of 60 participants was considered feasible for the BARITONE trial. To account for drop-out, the goal was to include 70 participants. The crossover design increases statistical efficiency by enabling within-individual comparisons of metabolite responses to dietary exposures. The BARITONE sample (*n* = 100–200) includes a separate evaluation sample (approximately *n* = 30) with habitual dietary intake data, which will be used to assess the robustness of the identified biomarker patterns.

### Dissemination policy

Results from the trial will be published on ClinicalTrials.gov as well as on the project homepage BARITONE-study|University of Gothenburg.

## Discussion

Dietary shifts toward more plant-based and diverse dietary patterns are urgently needed to mitigate risks to human health (e.g., cardio-vascular diseases and cancer) and to planetary health (e.g., climate change and environmental degradation). Our ability to accurately measure dietary intake is key to both advancing the nutrition science of diet and health associations and implementing effective public health strategies. Metabolomics offers the possibility to evaluate patterns of biomarkers and thus represents an objective dietary intake assessment method. Unfortunately, validated biomarkers for sources of protein and intake of vegetables, fruits, and berries, are still lacking. Here, a combination of controlled feeding studies and observational data on habitual intake has been called for.

The BARITONE project addresses this call by collecting detailed data on habitual dietary intake using a 7-day weighed food diary at a screening visit and a cross-over trial with different protein sources in a selected subsample from the screening population. During the three intervention weeks, the only difference in dietary intake is the protein source (meat, fish, or vegetarian, respectively). Blood and urine samples are collected prior to and after each intervention week, making it possible to evaluate changes in biomarker patterns within individuals related to protein source. Prediction models generated from these data will be validated using blood and urine samples and the habitual dietary intake data collected at the screening visit in the larger population.

Due to the lack of suitable comparator foods for vegetables, fruits, and berries, the only way to identify relevant biomarker patterns is to compare changes in the intake of these foods. Therefore, BARITONE participants are selected based on habitual high and low intake of vegetables, fruits, and berries, respectively, to address the second aim of the present study. Changes in biomarker patterns of these foods will be evaluated versus the trial diets and the 7-day weighed food records collected during the two 1-week washout periods between intervention weeks that reflect the habitual intake.

The project will enable identification of metabolomic biomarker patterns of key dietary components under controlled conditions and their validation in data on habitual dietary intake, while taking inter-individual variability into account.

### Strengths

Strengths of the BARITONE project include the detailed collection of dietary intake data, using 7-day weighed food diaries checked and compiled by registered dietitians. Participants undergo detailed phenotyping including blood, urine and feces sampling and body composition measurement. The trial is crossover as a way to minimize the effect of inter-individual variability in the assessment of biomarker responses to dietary intake.

Few metabolomic studies evaluate food intake in short-term interventions reflecting present intake. Food biomarkers measured postprandially are useful for identifying food-derived metabolites. In contrast, metabolites measured in fasting samples reflect present intake and this is likely related to health effects. Fasting samples are most commonly collected in epidemiological research. Hence, the results of our intervention study will be useful for interpreting metabolites sampled in larger epidemiological studies.

All intervention foods are provided free of charge. The provision of foods, together with simple standardized recipes and the use of selected convenience products where appropriate, are intended to facilitate adherence. Prespecified food products are used whenever available to reduce variation in food composition and any necessary product substitutions, such as a change of meatball product due to availability, are documented. Participants receive standardized recipes, specified quantities and detailed preparation instructions to minimize variation in cooking and preparation. Furthermore, the 7-day weighed food diaries completed during the washout periods provide detailed information on the types and amounts of foods consumed before each intervention period. This facilitates the interpretation of discriminating metabolites and the assessment of whether they may reflect the intervention foods, preceding dietary intake or both.

### Limitations

Some limitations must be acknowledged. The BARITONE participants do not represent a population-based sample but rather a self-selected group with special interest in diet and health. Further, the BARITONE trial participants constitute a subset of the larger BARITONE sample population and this reduces the inter-individual differences between the datasets, thus affecting the robustness test of the prediction algorithms within BARITONE. An external validation in a different study therefore is planned.

The intervention targets broad food categories, including different sources of protein and a range of vegetables, fruits and berries. Individual foods within these categories may differ in their chemical composition and associated metabolite profiles, which may reduce the ability to identify biomarkers that are common to an entire food category. Although prespecified products, standardized quantities, recipes and preparation instructions are used to minimize variation, foods are obtained over an extended period and do not necessarily originate from the same production batches. Necessary product substitutions are documented. The intervention foods are not chemically analyzed, as this would require analysis of multiple batches and substituted products. Consequently, residual variation related to the specific food item, natural and batch-to-batch variation, product substitution and participant preparation cannot be quantified. Furthermore, estimates based on food-composition databases are subject to limitations in database coverage and may not precisely represent the composition of the products consumed.

The study aims to identify biomarker patterns for food groups rather than for single foods. However, certain foods have unique characteristic metabolites that may differ between different compositions within a given food group. This could potentially limit the generalizability of the identified metabolite patterns to diets similar to the intervention diets or foods habitually consumed by the study participants. However, the results will later be validated also in other external cohorts.

## Conclusion

The BARITONE project will enable identification of metabolomic biomarker patterns of key dietary components needed to enhance the transition to a more plant-based dietary intake pattern. The BARITONE trial is conducted under controlled conditions taking inter-individual variability into account and the prediction models of biomarker patterns will be validated using detailed data on habitual dietary intake.

## Data Availability

The original contributions presented in the study are included in the article/Supplementary material, further inquiries can be directed to the corresponding author.
